# Genetic determinants restricting the reassortment of heterologous NSP2 genes into the simian rotavirus SA11 genome

**DOI:** 10.1038/s41598-017-08068-w

**Published:** 2017-08-24

**Authors:** Rebecca Mingo, Shu Zhang, Courtney P. Long, Leslie E. W. LaConte, Sarah M. McDonald

**Affiliations:** 1 Virginia Tech Carilion School of Medicine and Research Institute, Roanoke, VA USA; 2 Translational Biology, Medicine, and Health Graduate Program, Roanoke, VA USA; 3 Department of Biomedical Sciences and Pathobiology, Virginia-Maryland College of Veterinary Medicine, Blacksburg, VA USA

## Abstract

Rotaviruses (RVs) can evolve through the process of reassortment, whereby the 11 double-stranded RNA genome segments are exchanged among strains during co-infection. However, reassortment is limited in cases where the genes or encoded proteins of co-infecting strains are functionally incompatible. In this study, we employed a helper virus-based reverse genetics system to identify NSP2 gene regions that correlate with restricted reassortment into simian RV strain SA11. We show that SA11 reassortants with NSP2 genes from human RV strains Wa or DS-1 were efficiently rescued and exhibit no detectable replication defects. However, we could not rescue an SA11 reassortant with a human RV strain AU-1 NSP2 gene, which differs from that of SA11 by 186 nucleotides (36 amino acids). To map restriction determinants, we engineered viruses to contain chimeric NSP2 genes in which specific regions of AU-1 sequence were substituted with SA11 sequence. We show that a region spanning AU-1 NSP2 gene nucleotides 784–820 is critical for the observed restriction; yet additional determinants reside in other gene regions. *In silico* and *in vitro* analyses were used to predict how the 784–820 region may impact NSP2 gene/protein function, thereby informing an understanding of the reassortment restriction mechanism.

## Introduction

A shared feature of viruses with segmented RNA genomes is their capacity to undergo reassortment during host cell co-infection^1^. When two or more viral strains co-infect the same cell, they can exchange their genome segments. This process results in reassortant progeny with genes derived from multiple parental strains. In some cases, the new combination of genes confers a selective advantage to the virus, for instance by allowing it to evade the host immune response or infect a new host species^1–4^. Because of its substantial impact on viral evolution, reassortment is thought to occur frequently for segmented RNA viruses, such that heterogeneous pools of viruses exist with seemingly random gene combinations. However, the prevalence of reassortants in most viral populations tends to be much lower than what is expected based upon chance combination alone, and some strains are incapable of reassorting genome segments with each other even during experimental co-infection of cultured cells or animals. These observations suggest that reassortment requires genetic and biochemical compatibility. Therefore, nucleotide and/or amino acid differences between parental (i.e., co-infecting) strains may function as determinants of reassortment restriction. Studies seeking to identify and characterize these determinants for a given segmented RNA virus will not only enhance an understanding of the factors tempering or promoting its evolution, but they will also shed light on the basic biology of its replication cycle.

Rotaviruses (RVs) are 11-segmented, double-stranded (ds) RNA viruses that infect a wide array of mammalian and avian hosts^5^. More than 8 genetically-divergent species of RVs have been identified in nature (groups A–H, and tentative group I), though the vast majority of human disease is caused by group A strains^5–8^. While inter-species reassortment (e.g., between group A and B strains) has never been documented, it was generally assumed that reassortment occurs readily among group A strains^9^. However, this notion has been challenged in the past decade, mainly by the results of large-scale comparative genomic studies of wildtype group A human and animal strains^10–21^. Together, these studies demonstrate that group A RVs maintain very specific and stable genome segment constellations (i.e., gene sets) despite co-circulating in the same communities and even co-infecting the same host. For instance, group A human strains generally belong to one of two major genogroups (Wa-like or DS-1-like) or to the minor AU-1-like genogroup^22^. Inter-genogroup reassortants are rarely identified in clinical fecal specimens from normal, otherwise healthy children presenting symptoms of gastroenteritis. Reassortants between group A human and animal strains are also rarely identified in clinical specimens, though there are reports of such strains isolated sporadically from humans, often those with confounding medical conditions, such as an immune-compromised status^2^. Low co-infection rates could certainly contribute to the low frequency of reassortants in nature. Genetic and biochemical incompatibilities between the genes (or encoded proteins) of parental strains also plays an important role. In support of this notion, the number of reassortants is consistently lower than what is expected based upon unrestricted combination following experimental co-infection of cultured cells with group A human and animal strains, and there is a non-random association of genome segments in the reassortants that do arise^23–25^. Thus, nucleotide and/or amino acid differences between group A parental strains are putative determinants of reassortment restriction.

Each of the 11 dsRNA genome segments of RV is organized as a central open-reading frame (ORF) flanked by 5′ and 3′ untranslated regions (UTRs)^5^. The mechanism of reassortment for RV is a natural by-product of the manner in which the genome segments are selectively packaged during the viral replication cycle. While this process is poorly understood, the evidence to date is most consistent with a model wherein one of each of the 11 genome segments is packaged into an assembling virion as a single-stranded, positive-sense RNA (+RNA) precursor^26^. More specifically, the 11 distinct RV +RNAs are thought to engage with each other via RNA-RNA interactions to form an assortment complex. The *cis*-acting packaging signals required for these selective RNA-RNA interactions are predicted to reside in the 5′ and 3′ termini of each segment but have not been determined in detail^27, 28^. During or subsequent to encapsidation of the assortment complex, the 11 +RNAs are converted into 11 dsRNA genome segments by the VP1/VP2 viral polymerase complex^29^. Reassortment, therefore, occurs when the +RNAs of one parental strain are displaced by cognate +RNAs of another parental strain in the assortment complex. Given this mechanism, it is not surprising that strain-specific nucleotide differences in the genome segments (i.e., +RNAs) of co-infecting parental strains might restrict reassortment at the level of assortment complex formation, encapsidation, or dsRNA synthesis. Such direct restrictions on reassortment within the co-infected cell may explain why strains belonging to different RV groups do not seem to reassort with each other. There is little to no inter-group sequence homology at the 5′ and 3′ termini of genome segments, which may contribute to failure of RNA-RNA interactions during assortment complex formation. However, for the more closely-related group A RVs with conserved 5′ and 3′ termini, reassortment restriction is more likely to occur in a different, indirect manner. Most group A RV reassortants are likely generated during co-infection, but they would not emerge at appreciable levels in the viral population if they contain gene/protein sets that interact sub-optimally and function poorly together.

In the current study, we sought to investigate determinants of reassortment restriction by employing a previously-described, helper virus-based reverse genetic system^30, 31^. This system allows for the targeted reassortment of the NSP2 genome segment (hereafter referred to as the NSP2 gene) into the genetic backbone of simian RV strain SA11. Using this approach, we rescued SA11 reassortants containing NSP2 genes from human RVs Wa or DS-1. However, we could not rescue an SA11 reassortant containing an NSP2 gene from the human strain AU-1, which differs from that of SA11 by 183 nucleotides (36 amino acids). A gain-of-function chimeric mutagenesis approach was used to show that at least some of the key restriction determinants map to a region of the NSP2 gene ORF spanning nucleotides 784–820. We show that the 12 nucleotide differences in this region did not alter the predicted folding of the NSP2 + RNA nor did they affect its capacity to serve as an efficient replication template *in vitro*. Yet, the 7 amino acid differences in this region were predicted to cause changes in NSP2 protein structural dynamics. Altogether, the results of this manuscript inform an understanding of RV reassortment restriction determinants and functional domains within the NSP2 + RNA/protein.

## Results and Discussion

### Rescue of simian RV reassortants with NSP2 genes derived from human RVs Wa and DS-1

**Figure 1:**
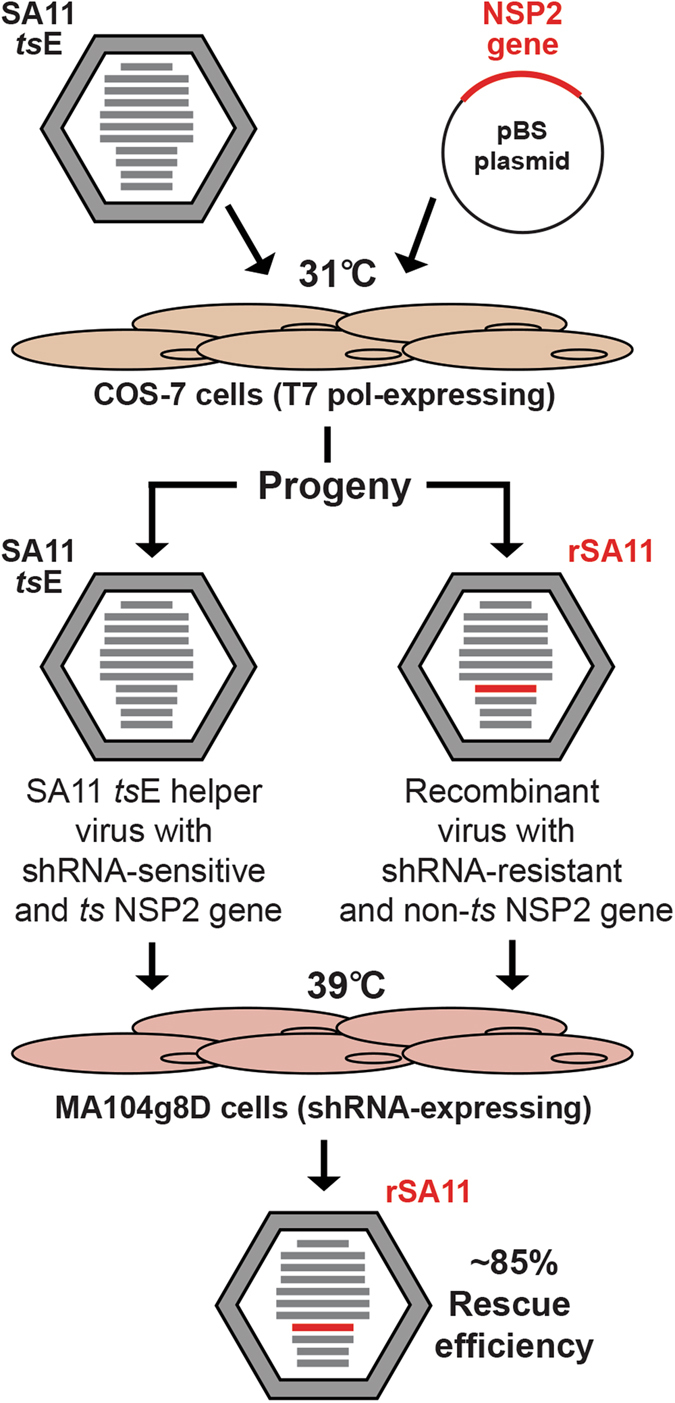
Overview of helper virus-based reverse genetics for the NSP2 gene. Cos-7 cells expressing T7 polymerase were transfected with the pBS plasmid that encodes an NSP2 + RNA. Following infection of the cells with the temperature-sensitive (*ts*) helper RV (SA11-*ts*E) at 31 °C, the plasmid-derived NSP2 + RNA (red) is reassorted into some of the viral progeny, making them recombinant (rSA11). Recombinants viruses are engineered to have an NSP2 gene that is resistant to shRNA degradation and encodes a non-temperature sensitive (*ts*) protein. Following two rounds of growth at 39 °C in shRNA-expressing MA104g8D cells, growth of SA11-*ts*E is suppressed and rSA11 is selected for with an efficiency of ~85% for the wildtype control.

**Figure 2:**
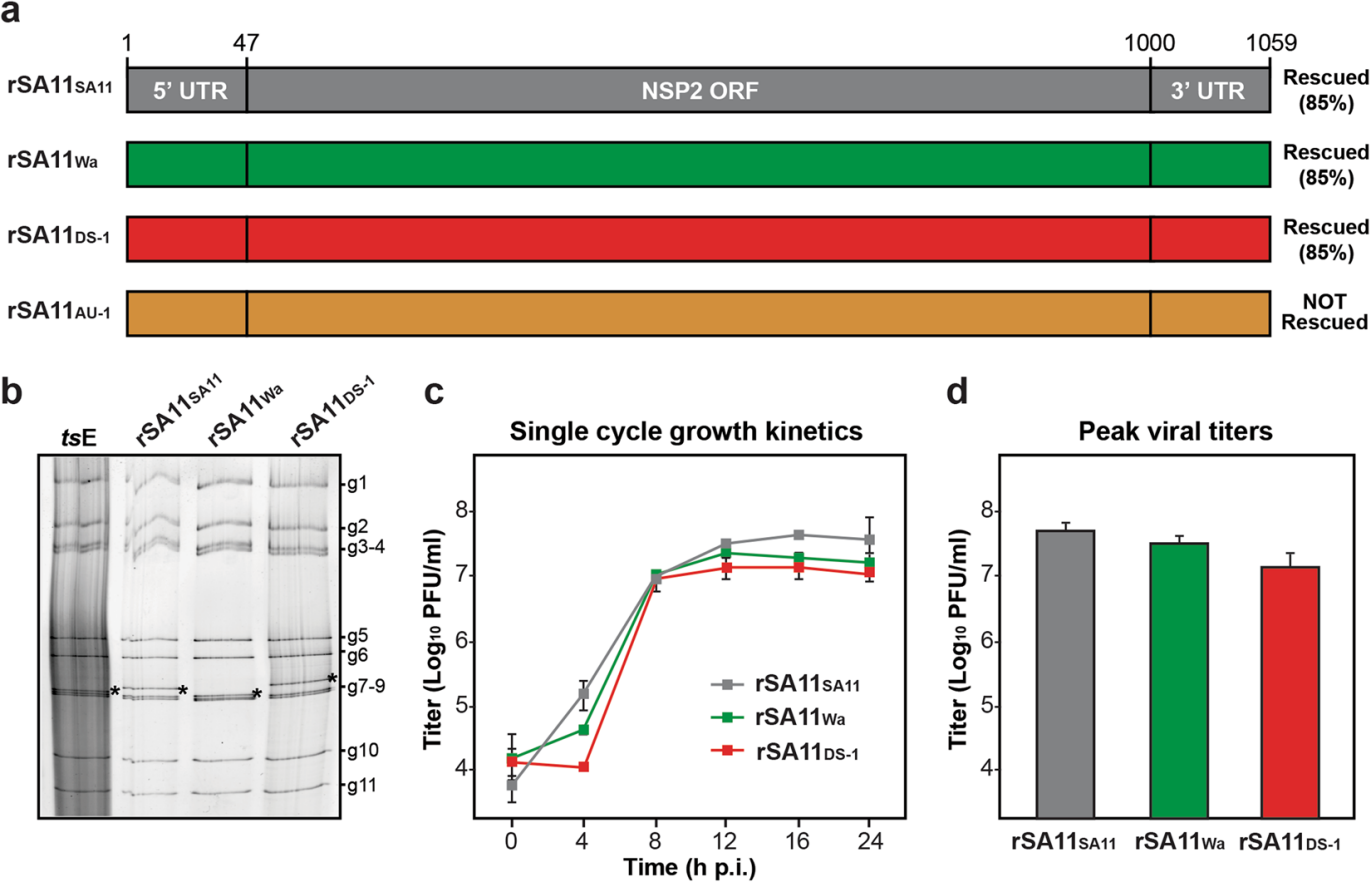
SA11 reassortants with human RV NSP2 genes. (**a**) NSP2 genes of recombinant reassortant viruses rSA11_SA11_, rSA11_Wa_, rSA11_DS-1_, and rSA11_AU-1_ are shown as boxes (not drawn to scale) and colored grey, green, red, and orange, respectively. Nucleotide numbers are listed at the top and the general locations of untranslated regions (UTRs) and open-reading frame (ORF) in the NSP2 gene are shown. The results of the rescue experiments, including % recombinant plaques, are listed to the right of each gene. (**b**) Electropherotyping. Viral dsRNA was extracted from rescued reassortants rSA11_SA11_, rSA11_Wa_, rSA11_DS-1_ and visualized following SDS-PAGE and SYBER Gold staining. The positions of the 11 viral genome segments are labeled to the right of the gel. The asterisk (*) shows the location of the reassorted NSP2 gene in each virus. (**c**) Single cycle growth kinetic assay. MA104 cells were infected at an MOI of 5 PFU per cell with rSA11_SA11_, rSA11_Wa_, or rSA11_DS-1_ and viral titers were determined at various hours (**h**) p.i. by plaque assay. The results of a single representative experiment are shown. Error bars represent standard deviation from the mean calculated from 3–4 plaque assay wells. (**d**) Peak viral titers. The graph shows average peak viral titers for rSA11_SA11_, rSA11_Wa_, or rSA11_DS-1_. Error bars represent standard deviation from the mean from two experiments (6–8 plaque assay wells). The differences in peak titers of rSA11_SA11_, rSA11_Wa_, or rSA11_DS-1_ were not statistically significant.

To investigate determinants of RV reassortment restriction, we employed a helper virus-based reverse genetics system that was developed by Trask *et al*.^30^ (Fig. 1). Specifically, we tested whether simian RV strain SA11 reassortants could be rescued with NSP2 genes derived from human RV genogroup prototype strains Wa, DS-1, or AU-1 (Fig. 2a). Monkey kidney Cos-7 cells were transfected with plasmids expressing the cloned human RV NSP2 genes as +RNAs (Fig. 1). The cells were then infected with simian RV strain SA11-*ts*E for 24 h at 31 °C to allow the cDNA plasmid-derived +RNAs to reassort into the viral genome. The cloned NSP2 +RNAs were engineered to be resistant to the g8D small hairpin (sh) RNA, and they encode a non-temperature-sensitive NSP2 protein. In contrast, the SA11-*ts*E NSP2 + RNA is g8D shRNA-sensitive and encodes a protein that is non-functional at high temperature^32^. Recombinant reassortant viruses generated in Cos-7 cells were selected for by two rounds of growth at 39 °C in g8D shRNA-expressing monkey kidney MA104 cells (Fig. 1). Plaque assays were then performed to isolate individual viral clones, and % rescue efficiency was calculated from the ratio of the number of clones identified as recombinant over the total number of clones investigated by RT-PCR and nucleotide sequencing.

We found that recombinant SA11 reassortants containing the wildtype SA11 NSP2 gene (rSA11_SA11_) and those containing the NSP2 genes from human RVs Wa or DS-1 (rSA11_Wa_ and rSA11_DS-1_, respectively) were rescued with 85% efficiency (Fig. 2a). In contrast, we were unable to rescue an SA11 reassortant with the human RV AU-1 NSP2 gene (rSA11_AU-1_), despite screening 100 plaques (Fig. 2a). The reassortant clones that we did rescue (rSA11_SA11_, rSA11_Wa_, and rSA11_DS-1_) were triple plaque-purified, amplified by two rounds of growth in MA104 cells, and their identities were confirmed by electropherotyping and complete NSP2 gene sequencing (Fig. 2b and data not shown). The sequences of the NSP2 genes from the rescued viruses were identical to the cDNA clones, suggesting that the recombinant viruses were genetically stable for at least ~5 passages (data not shown). To test whether rSA11_Wa_ or rSA11_DS-1_ exhibit replication defects, single cycle growth kinetic assays were performed in MA104 cells (Fig. 2c). The results show that both rSA11_Wa_ and rSA11_DS-1_ replicated as well as the isogenic control virus, rSA11_SA11_, with no statistically-significant differences in peak titers (Fig. 2d). When compared to the SA11 NSP2 gene, that of Wa differs by 198 nucleotides (41 amino acids) and that of DS-1 differs by 225 nucleotides (45 amino acids) (Fig. S1). Based on the efficient rescue and growth of rSA11_Wa_ and rSA11_DS-1_, we conclude that these differences do not represent reassortment restriction determinants into the SA11 genetic backbone. However, the lack of rSA11_AU-1_ rescue suggests that (i) the AU-1 NSP2 + RNA was not efficiently packaged/replicated by the SA11-*ts*E helper virus and/or that (ii) the AU-1 NSP2 + RNA/protein is not functionally compatible with the other SA11 +RNAs/proteins during the subsequent *de novo* viral replication that is required for plaque formation.

### Mapping reassortment restriction determinants in the AU-1 NSP2 gene

**Figure 3:**
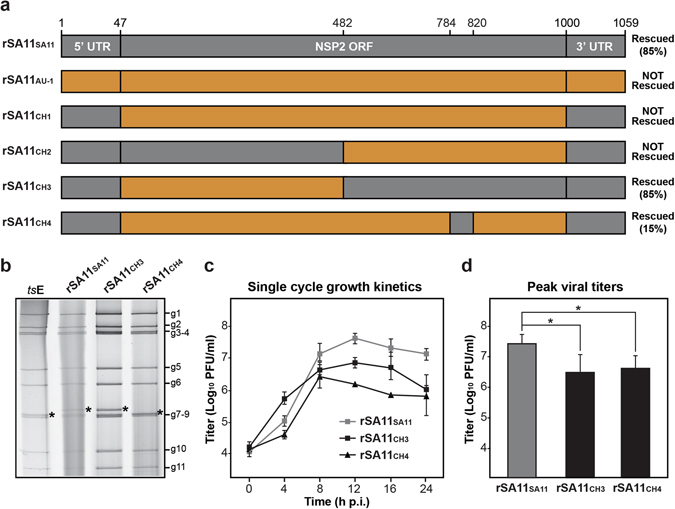
SA11 reassortants with chimeric NSP2 genes. (**a**) NSP2 genes of recombinant reassortant viruses are shown as boxes (not drawn to scale). For all genes, SA11 sequence is shown in grey and AU-1 sequence is shown in orange. Nucleotide numbers are listed at the top and the general locations of untranslated regions (UTRs) and open-reading frame (ORF) in the NSP2 gene are shown. The result of the rescue experiments, including % recombinant plaques, are listed to the right of each gene. (**b**) Electropherotyping. Viral dsRNA from was extracted from rescued reassortants rSA11_SA11_, rSA11_CH3_, rSA11_CH4_ visualized following SDS-PAGE and SYBER Gold staining. The positions of the 11 viral genome segments are labeled to the right of the gel. The asterisk (*) shows the location of the reassorted NSP2 gene in each virus. (**c**) Single cycle growth kinetic assay. MA104 cells were infected at an MOI of 5 PFU per cell with rSA11_SA11_, rSA11_CH3_, or rSA11_CH4_ and viral titers were determined at various hours (**h**) p.i. by plaque assay. The results of a single representative experiment are shown. Error bars represent standard deviation from the mean calculated from 3–4 plaque assay wells. (**d**) Peak viral titers. The graph shows average peak titer for rSA11_SA11_, rSA11_CH3_, or rSA11_CH4_. Error bars represent standard deviation from two experiments (6–8 plaque assay wells). Asterisks (*) indicate statistically significant differences (*p* < 0.05).

The AU-1 NSP2 gene differs from that of SA11 by 183 nucleotides (36 amino acids) (Fig. S1). To narrow down which of these differences correlate with AU-1 NSP2 gene reassortment restriction into SA11, we employed a gain-of-function chimeric mutagenesis approach. Specifically, we cloned AU-1 NSP2 genes that contained the SA11 5′ and 3′ UTRs as well as SA11 sequence fragments in various ORF regions (Fig. 3a). We tested whether we could rescue SA11 reassortants using these chimeric NSP2 genes. We found that we were unable to rescue reassortant rSA11_CH1_, despite the fact that this NSP2 gene has authentic SA11 UTRs. This result suggests that at least some of the restriction determinants map within the gene ORF. Likewise, we could not rescue rSA11_CH2_, which contains SA11 UTRs as well as SA11 sequence in an ORF region spanning nucleotides 47–481. Thus, the downstream region of 482–1000 must contain important restriction determinants. In support of this notion, we were able to rescue rSA11_CH3_ and rSA11_CH4_ with efficiencies of 85% and 15%, respectively. The reassortant rSA11_CH3_ virus contains an AU-1 NSP2 ORF in which nucleotides 482–1000 were replaced with SA11 sequence. The reassortant rSA11_CH4_ virus contains an AU-1 NSP2 ORF in which only a smaller sub-region (SR) corresponding to nucleotides 784–820 was replaced with SA11 sequence. That rationale for changing only the SR was based upon the observation that 36% of the coding changes clustered in this 37-nucleotide stretch of the NSP2 ORF.

**Figure 4:**
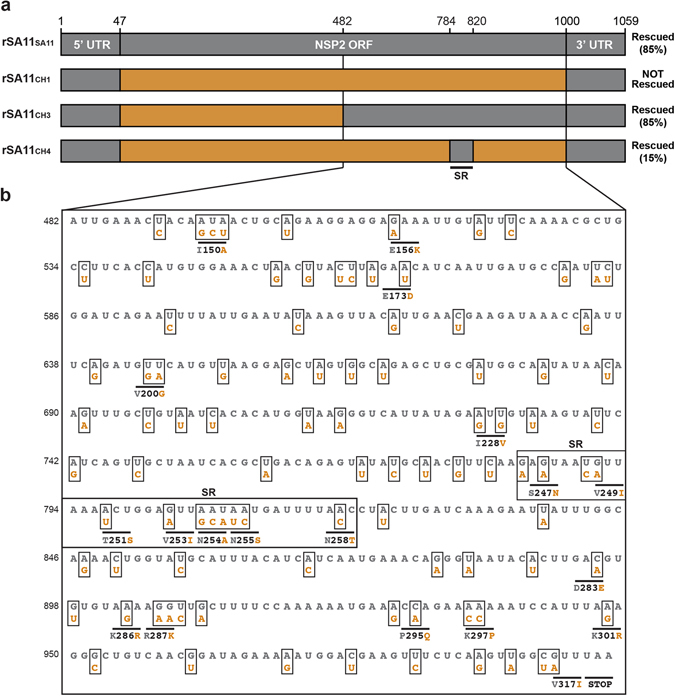
Nucleotide and amino acid differences between SA11 NSP2 and AU-1 NSP2 in the 482–1000 region. (**a**) NSP2 genes of recombinant reassortant viruses with wildtype SA11 or chimeric NSP2 genes are shown as boxes (not drawn to scale). For all genes, SA11 sequence is shown in grey and AU-1 sequence is shown in orange. Nucleotide numbers are listed at the top and the general locations of untranslated regions (UTRs) and open-reading frame (ORF) in the NSP2 gene are shown. The results of the rescue experiments, including % recombinant plaques, are listed to the right of each gene. (**b**) The sequence of nucleotides 482–1000 of SA11 NSP2 are shown in grey. The nucleotides that differ for AU-1 NSP2 are shown below the SA11 sequence and are colored orange. Amino acid changes are listed beneath the corresponding codon with the SA11 residue shown in grey and the AU-1 residue shown in orange. The SR (nucleotides 784–820) is outlined with a box and labeled.

The reassortant clones (rSA11_CH3_ and rSA11_CH4_) were triple plaque-purified, amplified by two rounds of growth in MA104 cells, and their identities were confirmed by electropherotyping and complete NSP2 gene sequencing (Fig. 3b and data not shown). The sequences of the NSP2 genes from the rescued viruses were identical to the cDNA clones, suggesting that the recombinant viruses were genetically stable at least for ~5 passages (data not shown). To test whether rSA11_CH3_ or rSA11_CH4_ exhibit replication defects, single cycle growth kinetic assays were performed in MA104 cells (Fig. 3c). The results show that both rSA11_CH3_ and rSA11_CH4_ had lower titers than the control rSA11_SA11_ virus at several times p.i. and both had statistically-significant lower peak viral titers (approximately 1 log_10_ step) compared to rSA11_SA11_ (Fig. 3d). This result suggests that both rSA11_CH3_ and rSA11_CH4_ have modest replication defects. However, the observation that that rSA11_CH4_ was rescued at all, despite its replication defect, indicates that the SR (nucleotides 784–820) contains important determinants of AU-1 NSP2 gene reassortment restriction. Still, the higher rescue efficiency of rSA11_CH3_ compared to rSA11_CH4_ (i.e., 85% v. 15%) indicate that additional residues outside the SR but within the larger 482–1000 region are important for robust NSP2 gene reassortment. When comparing the NSP2 gene sequences of SA11 vs. AU-1, we found a total of 95 nucleotide (19 amino acid) differences in the 482–1000 region, and only 12 nucleotide (7 amino acid) differences reside within the SR (Fig. 4). At this time, we cannot exclude the possibility that the UTRs of the AU-1 NSP2 gene also contain restriction determinants when appended to the SA11 ORF. This is especially true for the 3′ UTR of the AU-1 NSP2 gene, which shows 7 nucleotide differences when compared to that of SA11; the 5′UTR only has a single nucleotide change and is unlikely to have a dramatic impact on reassortment restriction (Fig. S1).

### Predicting the contributions of the 12 SR nucleotide changes on the NSP2 + RNA

**Figure 5:**
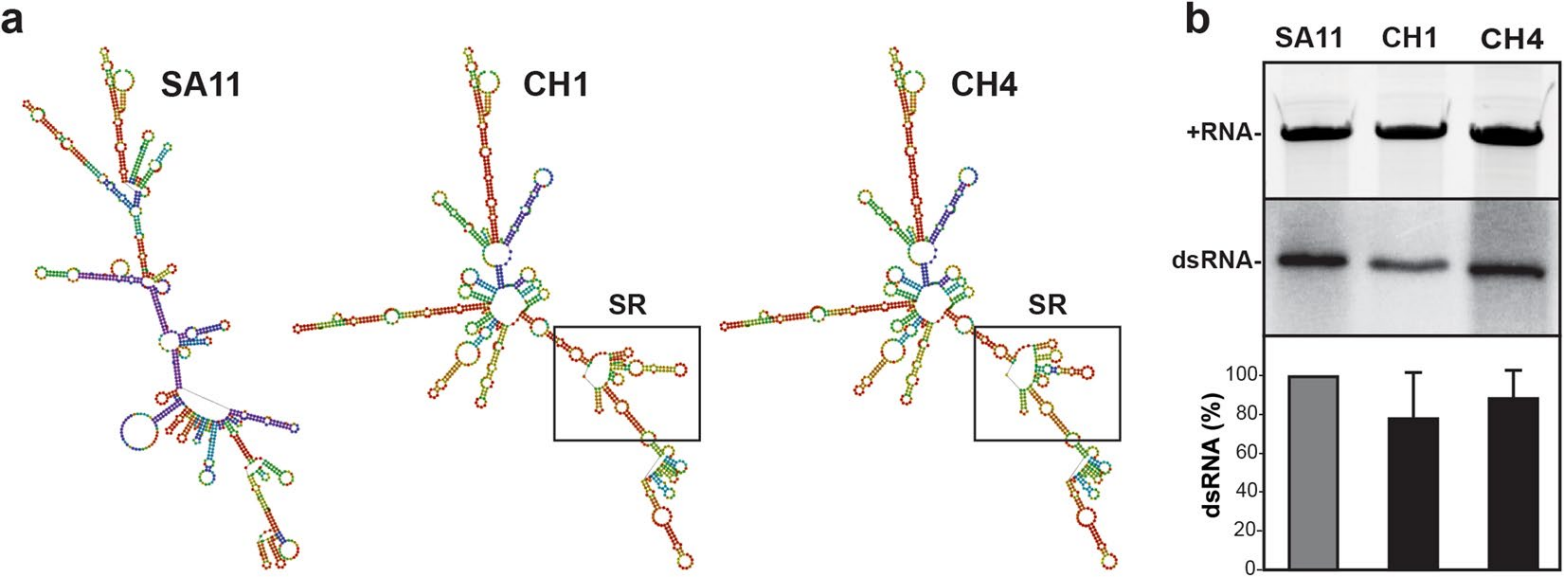
Predicted folding and *in vitro* replication of NSP2 +RNAs. (**a**) The predicted secondary structures of SA11, CH1, and CH4 +RNAs are shown and colored according to base-pair probabilities (warmer color = more favorable, cooler color = less favorable). The nucleotides comprising the SR of CH1 and CH4 + RNA is outlined with a box. (**b**) *In vitro* replicase assays. The SA11, CH1, and CH4 + RNA templates were electrophoresed in a 7M-urea-acrylamide gel and visualized following ethidium bromide staining (top gel panel). Gel image was cropped to show only the + RNA bands. These +RNAs were then assayed for their capacity to serve as templates for dsRNA synthesis by SA11 VP1/VP2 *in vitro*. The nascent minus-strands of the dsRNAs were radiolabeled using [^32^P]αUTP and detected following SDS-PAGE and autoradiography (bottom gel panel, representative experiment). Gel image was cropped to show only the dsRNA bands. The dsRNA levels were quantified for 4 independent experiments and shown as a % relative to SA11 dsRNA. The differences in CH1 and CH4 dsRNA levels relative to SA11 are not statistically significant.

We next sought to gain insight into how the 12 SA11-specific nucleotides in the SR might have increased the efficiency of the AU-1 NSP2 gene reassortment into the SA11 genetic backbone in the context of rSA11_CH4_ allowing rescue using the reverse genetics system. We reasoned that the NSP2 gene + RNA of rSA11_CH4_ (CH4 + RNA) might show differences in its folding or function relative to that of rSA11_CH1_ (CH1 + RNA), which was not rescued in our experiments and lacks the 12 SA11-specific SR nucleotides, but maintains the SA11 UTRs (Fig. 3a). To assess this possibility, we predicted the minimum free energy secondary structures and base pair probabilities of the +RNAs *in silico* (Fig. 5a). The results show that CH1 and CH4 +RNAs are predicted to have similar overall folds with only minor energetic changes in the SR. To test whether CH1 and CH4 +RNAs have differences in their capacity to serve as templates for replication, we employed an *in vitro* dsRNA synthesis assay (Fig. 5b). We found that CH1 and CH4 NSP2 +RNAs were efficiently converted into dsRNA products by SA11 VP1/VP2 at levels comparable to the wildtype SA11 NSP2 + RNA control. Thus, the NSP2 + RNA of the non-rescued rSA11_CH1_ virus is indistinguishable in its predicted folding and *in vitro* replicative capacity from that of the rescued rSA11_CH4_ virus. This result suggests that the difference in rescue efficiency/viral growth between rSA11_CH1_ and rSA11_CH4_ cannot be easily explained at the + RNA level. Still, it remains possible that the CH1 + RNA has defects in RNA-RNA interactions required for assortment/packaging into SA11, which we are unable to experimentally test using this helper virus-based reverse genetics system.

### Predicting the contributions of 7 SR amino acid changes on NSP2 protein structural dynamics

NSP2 is a 35-kDa viral protein that forms a stable, donut-shaped octamer^32, 33^. NSP2 interacts with viral RNA and four viral proteins (VP1, VP2, NSP5 and NSP6), and it is essential for RV particle assembly and dsRNA synthesis^34–39^. One possible reason for the lack of rescuable AU-1 and CH1 NSP2 gene reassortants in our reverse genetics experiments could be that the AU-1 NSP2 protein does not functionally interact or interacts only sub-optimally with its viral binding partners (or with as of yet unknown cellular binding partners). In this case, even if the reassortment event occurred in the transfected-infected Cos-7 cells, the rSA11_AU-1_ and rSA11_CH1_ viruses would not replicate to detectable levels in MA104g8D cells. By changing the 7 AU-1-specific amino acid residues of the SR to match those of SA11 in the context of rSA11_CH4_, we may have restored the capacity of the NSP2 protein to better interact with its binding partners, thereby improving replication enough to rescue the virus.

**Figure 6:**
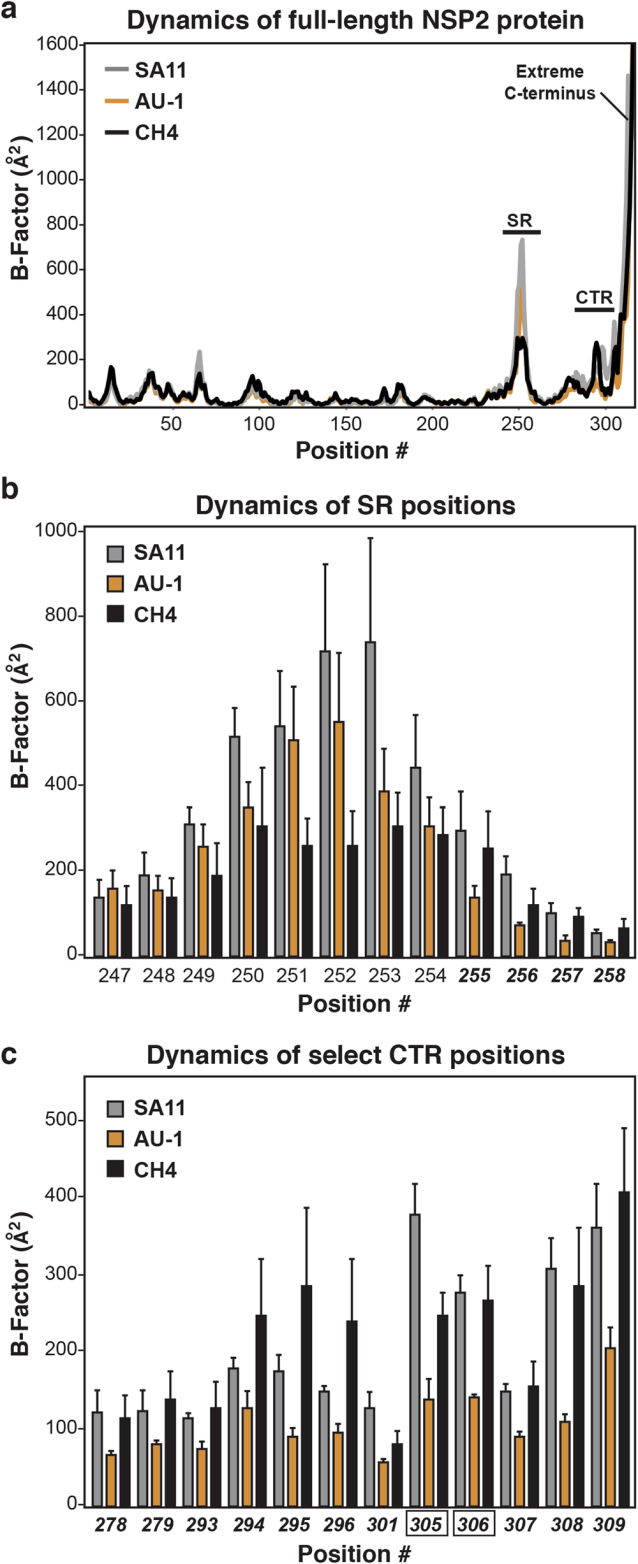
Molecular dynamics simulations of NSP2 proteins. (**a**) Average B-factors are shown for each position of SA11 NSP2 (grey), AU-1 NSP2 (orange), and CH4 NSP2 (black). The regions corresponding to the SR, CTR, and extreme C-terminus of the NSP2 protein are labeled. Dynamics of the (**b**) SR positions and (**c**) select CTR positions. The graphs indicate relative flexibility at individual positions for SA11 NSP2 (grey), AU-1 NSP2 (orange), and CH4 NSP2 (black). Error bars represent standard deviation from the mean following 3 independent simulations. The positions numbers in bold-italic type are those in which the predicted B-factors of CH4 NSP2 are similar to those of SA11 NSP2. Positions 305 and 306 (black box outline) showed statistically-significant differences (*p* < 0.05) when comparing B-factors for SA11 v. AU-1 NSP2 and AU-1 v. CH4 NSP2.

To gain insight into possible differences in the NSP2 protein as a result of the 7 SR amino acid changes, we performed unrestrained molecular dynamics simulations. Specifically, the monomeric structures of SA11 NSP2, AU-1 NSP2, and CH4 NSP2 were each simulated for 20 nanoseconds at 300 K. Root mean square fluctuations (RMSFs) of the α-carbons were used to calculate B-factors, thereby providing an estimate of the degree of flexibility at each position of the protein. Several regions of flexibility were predicted in all three NSP2 proteins (Fig. 6a). In particular, the SR (positions 247–258) is a flexible loop element, and it is followed by a flexible C-terminal region (CTR; positions Change “258” to “275”–311). The extreme C-terminus (positions 312–317) showed dramatic, but consistent, movement for all three protein structures. Movement of residues in and around the SR (i.e., positions 235–243 and 251–256) have been described to occur following RNA binding of NSP2^34^. Moreover, flexibility of the NSP2 C-terminus has been previously reported, and it is suggested to be important for domain-swapping interactions that link NSP2 octamers together^37^.

We reasoned that the structural dynamics of CH4 NSP2 would largely match those of AU-1 NSP2, except at sites that were functionally restored due to the 7 SR amino acid changes. We thought that those restored sites of CH4 NSP2 would show flexibility similar to that observed for SA11 NSP2. In particular, we predicted that the relative flexibility of the SR would be the same for CH4 NSP2 and SA11 NSP2 because they are the same sequence. Surprisingly, we found that most SR positions exhibited lower B-factors for CH4 NSP2 compared to both SA11 and AU-1 NSP2 proteins (Fig. 6b). The flexibility at only SR positions 255–258 were similar between CH4 NSP2 and SA11 NSP2, but differed for that of AU-1 NSP2 (Fig. 6b). We also identified 12 positions in the CTR (278–279, 293–296, 301, and 305–309) for which CH4 NSP2 flexibility matched that of SA11 NSP2, but not AU-1 NSP2 (Fig. 6c). While general trends in the data were observed, only positions 305 and 306 showed statistically significant differences in B-factors for both SA11 v. AU-1 NSP2 as well as AU-1 v. CH4 NSP2. Still, the decreased B-factors observed at all 16 positions in AU-1 NSP2, which did not allow for rescue of an SA11 reassortant, are indicative of a more ordered structure that is potentially restricted in interacting with binding partners or undergoing conformational change. Thus, the restoration of motion in CH4 NSP2 in the SR and CTR may be essential for optimal NSP2 interactions with binding partners, thereby contributing to the rescue of the rSA11_CH4_ virus.

**Figure 7:**
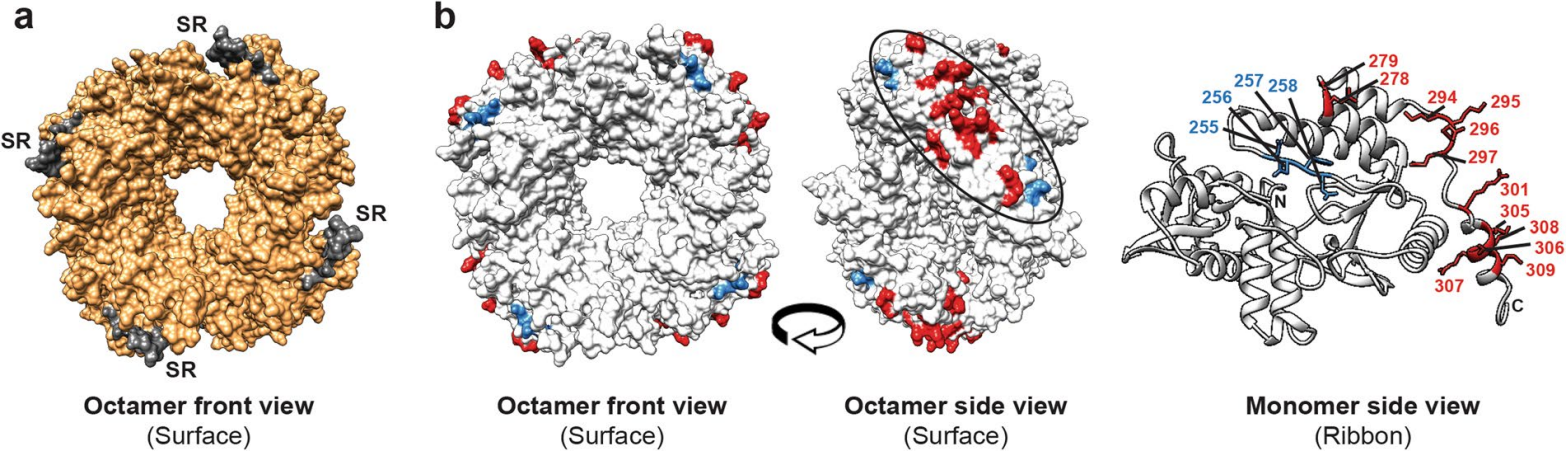
Location of dynamically-restored residues on the CH4 NSP2 protein structure. (**a**) Homology model of CH4 NSP2 is shown as an octamer (surface-filled and viewed from the front). Regions of the protein corresponding to AU-1 NSP2 sequence are shown in orange and those of SA11 sequence (i.e., the SR) are shown in grey. (**b**) Left: The CH4 NSP2 octamer is shown in the same position as panel A but colored white. The 4 restored positions that map to the SR are shown in blue and the 12 restored positions that map to the CTR are shown in red. Middle: These 16 positions form a large patch on the side of the octamer structure. Right: The restored positions are shown in stick representation and labeled on a single CH4 NSP2 monomer ribbon structure.

Using the homology model of CH4 NSP2 (Fig. 7a), we determined the three-dimensional location of the 16 positions that exhibited restored flexibility. These 16 positions comprise a large surface-exposed patch on the side of the octamer (Fig. 7b). The location of this patch is amenable to protein/RNA interactions, though it does not significantly overlap with any of the known or predicted binding sites on NSP2^34–39^. The molecular dynamics simulations and modeling results suggest that that amino acid changes in the NSP2 SR can alter the structural dynamics of the NSP2 CTR. Thus, this data raises questions about whether altering amino acids as a result of the SR swap alleviated proximal structure-based determinants of gene reassortment restriction for AU-1 NSP2 into SA11.

### Summary and conclusions

It has long been observed that reassortment is restricted among some group A RV strains; yet the genetic determinants that mediate such restriction are completely unknown. In the current study, we employed a helper virus-based reverse genetics system and attempted to engineer simian RV strain SA11 reassortants containing NSP2 genes from prototypic human RV strains Wa, DS-1, and AU-1 (rSA11_Wa_, rSA11_DS-1_, and rSA11_AU-1_, respectively). We showed that rSA11_Wa_ and rSA11_DS-1_ were efficiently rescued and exhibited no detectable growth defects. In contrast, rSA11_AU-1_ was not rescuable in this system. This result was surprising and led us to test whether we could overcome the observed reassortment restriction by replacing regions of the AU-1 NSP2 gene (i.e., that contain restriction determinants) with SA11 sequence (i.e., that lack restriction determinants). Using this approach, we found that restriction determinants map to the SR (nucleotides 784–820) within the AU-1 NSP2 gene ORF. However, it is important to note that the SR is not the *only* region containing restriction determinants; there are certainly other regions that contributed to the lack of rescuable rSA11_AU-1_ virus. In support of this notion, we found that rSA11_CH3_ had modest a replication defect, indicating that determinants of robust reassortant viral growth reside within ORF nucleotides 47–481. While not tested here, the 3′UTR of the AU-1 NSP2 gene may also contain restriction determinants. Nevertheless, the molecular details of exactly *how* any of these regions influence reassortment restriction is not understood. For the SR, we hypothesize that the restriction determinants do not prevent packaging of the AU-1 NSP2 + RNA into SA11 during the assortment process in Cos-7 cells. Instead, we think that it is more likely that the AU-1-specific SR nucleotides/amino acids reduced replication of the reassortant virus, thereby preventing its detection following growth in MA104g8D cells. Our *in vitro* and *in silico* analyses of the CH1 vs. CH4 +RNAs, which differ by 12 nucleotides, did not reveal any obvious differences that could account for the restriction being mediated at the RNA-level. In contrast, our *in silico* analyses of the NSP2 protein showed that the SR encodes a highly flexible and surface-exposed loop element. The 7 amino acid changes that resulted from the SR sequence swap are predicted to alter the local flexibility of the AU-1 NPS2 SR and CTR in a way that more closely resembles the that of SA11 NSP2. It is possible, albeit not experimentally validated, that this change in flexibility allowed the chimeric NSP2 protein, but not the AU-1 NSP2 protein, to engage viral or cellular binding partners, improving its replicative fitness. The SR is a highly variable site on the NSP2 protein (<50% sequence identity among group A RV strains). When comparing the SR sequences of strains SA11, Wa, DS-1, and AU-1, we find that AU-1 has unique residues at positions 249, 253–255, and 258, which may be the primary determinants for restriction in this case (Fig. S1). Ongoing and future work in our laboratory is employing biochemical approaches to test the hypothesis that restriction is mediated by sub-optimal NSP2-protein or NSP2-RNA interactions due to the identified amino acid changes.

During the writing of this manuscript, a plasmid only-based reverse genetics system was reported by Kanai *et al*., which allows for the targeted manipulation of any SA11 gene and rescue in the absence of a helper virus^40^. Without a doubt, this new system will provide an improved experimental platform for detailed investigations of RV gene reassortment restriction mechanisms, including the study of genetic epistasis among multiple segments. However, one advantage of the helper virus-based system that we used here is that it more closely mimics co-infection conditions. Much like in nature, only reassortants with at least a moderate degree of fitness will be rescued using the helper virus-based approach, because it requires that recombinant +RNAs successfully compete with those of the helper virus. It is possible that replication-defective reassortants will be rescued using the plasmid-only reverse genetic system because there is no competition of wildtype +RNAs during the assortment step; these same reassortants would not emerge from co-infected cells. On the other hand, the fully plasmid-based system will allow for the differentiation of direct restrictions (i.e., failure of + RNA packaging) vs. indirect restrictions (i.e., failure of +RNAs/proteins to interact during *de novo* replication). In essence, non-rescuable reassortants from the fully-plasmid system must represent a direct restriction that prevents the generation of hybrid progeny. Regardless of the genetic system used, studies seeking to uncover the molecular details that prevent reassortants from emerging in the RV population are warranted because they will inform rational infection control measures and elucidate new aspects of virus biology.

## Materials and Methods

### Cells and viruses

Monkey kidney cell lines (MA104 and Cos-7) were obtained from American Type Cell Culture and maintained as described by Arnold *et al*., 2009 in medium (Life Technologies) that was supplemented to contain 5–10% heat-inactivated fetal bovine serum, 100 U/ml penicillin, 100 µg/ml streptomycin, and 0.5 µg/ml Amphotericin B^41^. MA104g8D cells stably expressing a short hairpin RNA (shRNA) against the NSP2 gene were obtained from Dr. John Patton (University of Maryland, College Park) and cultured in the same manner as MA104 cells^30^. RV strains Wa, DS-1, and AU-1 were obtained from Dr. John Patton (University of Maryland, College Park). RV strain SA11-*ts*E (clone 1400) was obtained from Dr. John Patton with permission from Dr. Frank Ramig (Baylor College of Medicine)^42^. RV infections were performed as described previously^41^. Replication-defective vaccinia virus expressing T7 polymerase (strain rDI-T7pol) was obtained from Dr. John Patton (University of Maryland, College Park) with permission from Dr. Koki Taniguchi (Fujita Health University School of Medicine, Japan) and was propagated as described previously^31, 32^.

### Plasmid construction

A plasmid expressing the full-length, shRNA-resistant NSP2 gene from simian RV strain SA11 (pBS-SA11g8^R^) was obtained from Dr. John Patton (University of Maryland, College Park)^31^. To construct similar plasmids expressing the full-length NSP2 genes from human RV strains Wa (pBS-Wag8^R^), DS-1 (pBS-DS-1g8^R^), or AU-1 (pBS-AU-1g8^R^) RT-PCR and molecular cloning was used. Specifically, viral RNA was extracted from MA104 cells infected with Wa, DS-1, or AU-1 and used as template in RT-PCR reactions to produce cDNA. Primer-generated restrictions sites 5′ *NcoI* and 3′ *AfeI* allowed the cDNA products of the Wa, DS-1, or AU-1 NSP2 genes to be individually ligated into the pBS-SA11g8^R^ plasmid in place of the SA11 NSP2 gene. Site-directed mutagenesis was then used to engineer shRNA resistance in the AU-1 NSP2 gene. The cloned Wa and DS-1 NSP2 gene sequences are identical to what is reported in GenBank (accession numbers RO1NS35F and EF672580, respectively). The AU-1 NSP2 gene sequence is identical to what is reported in GenBank (accession number DQ490534) with the exception of a single G-to-A nucleotide change at position 40 in the 5′ UTR and the 6 nucleotide changes in the shRNA target site (nucleotides 655–673). The NSP2 proteins encoded by all three cloned genes are identical to GenBank sequences. To engineer the chimeric NSP2 genes, PCR was used to amplify specified regions (Fig. 3a) from the pBS-SA11g8^R^ and pBS-AU-1g8^R^ plasmids. The Golden Gate Assembly kit (New England Biolabs) was then used to seamlessly ligate the amplified cDNAs together, creating plasmids pBS-CH1g8^R^, pBS-CH2g8^R^, pBS-CH3g8^R^, and pBS-CH4g8^R^. Final plasmids were sequenced to ensure integrity of the gene and absence of PCR-induced mutations. All primers used for cloning are available by request.

### Generation and recovery of recombinant SA11 reassortants

Recombinant SA11 reassortants were generated as described previously^31, 32^. Briefly, Cos-7 cells in 6-cm tissue culture dishes (~1.25 × 10^6^ cells/well) were inoculated with rDI-T7pol at a multiplicity of infection (MOI) of 3 plaque-forming units (PFU) per cell and then transfected with 2.5 μg of plasmid DNA (pBS-SA11g8^R^, pBS-Wag8^R^, pBS-DS-1g8^R^, pBS-AU-1g8^R^, pBS-CH1g8^R^, pBS-CH2g8^R^, pBS-CH3g8^R^, or pBS-CH4g8^R^) using Trans-IT LT1 (Mirus). The cells were incubated at 37 °C for 24 hours to allow for + RNA expression from the plasmids, and then they were infected with the helper virus SA11-*ts*E at an MOI of 5 PFU per cell at 31 °C for 24 hours. Cells were harvested by 3 rounds of freeze-thaw, and then the clarified supernatant (P0 stock) was passaged in MA104g8D cells at 39 °C for two rounds (P1 and P2 stocks) in the presence of 10 μM AraC (Fisher) to inhibit rDI-T7pol replication^31^. Recombinant reassortants were detected in the P2 stocks by RT-PCR and sequencing of the NSP2 gene. For rSA11-NSP2_SA11_, rSA11-NSP2_Wa_, rSA11-NSP2_DS-1_ and rSA11-NSP2_CH3_, 17/20 plaques each were identified as recombinant (85% rescue efficiency). For rSA11-NSP2_CH4_, 3/20 plaques were identified as recombinant (15% rescue efficiency). For rSA11-NSP2_AU-1_, rSA11-NSP2_CH1_, and rSA11-NSP2_CH2_ no recombinant viruses were detected in the P2 stocks by RT-PCR/sequencing, nor from screening 20–100 individual plaques. Rescued recombinants were subjected to three rounds of plaque-purification in MA104 cells to isolate viral clones^41^. Final high-titer SA11 reassortant stocks were grown for two passages in MA104 cells, and they were verified by RT-PCR, sequencing of the NSP2 gene, and electrophoretic analysis of viral dsRNA as described previously^31^.

### Single cycle growth kinetic assays

MA104 cells were infected with the recombinant wildtype control virus (rSA11-NSP2_SA11_) or a rescued reassortant (rSA11-NSP2_Wa_, rSA11-NSP2_DS-1_, rSA11-NSP2_CH3_, or rSA11-NSP2_CH4_) at an MOI of 5 PFU per cell. At the indicated times p.i., cells were harvested by 3 rounds of freeze-thaw, and infectious virus in the clarified lysate was quantitated by plaque assay^41^. Titers at 4, 8, 12, 16 and 24 hours p.i. were normalized based upon the titers at 0 hours p.i. for each virus. Two-tailed tests were performed using Smith’s Statistical Software, and *p* values < 0.05 were considered to be statistically significant.

### Modeling of NSP2 + RNA folding and *in vitro* replicase assays

Minimum free energy secondary structure and base pair probabilities were calculated for SA11, CH1, and CH4 NSP2 +RNAs using the RNAfold webserver (http://rna.tbi.univie.ac.at/cgi-bin/RNAfold.cgi)^43^. The + RNA templates used in the *in vitro* replicase assays were prepared using the reverse genetic plasmids (pBS-SA11g8^R^, pBS-CH1g8^R^, and pBS-CH4g8^R^) and the protocol described by McDonald *et al*.^44^. Template quantity was determined by using a UV spectrophotometer (optical density at 260 nm [OD_260_]) and quality was assessed by electrophoresis in 7 M urea-5% polyacrylamide gels stained with ethidium bromide. SA11 VP1 and VP2 purifications and *in vitro* replicase assays were performed as described by McKell *et al*.^45^. Two-tailed tests were performed using Smith’s Statistical Software, and *p* values < 0.05 were considered to be statistically significant.

### Molecular dynamics simulations of NSP2 proteins

Molecular dynamics simulations using a standard approach^46^ were performed using GROMACS v5.1.3^47^ on the atomic structure of the SA11 NSP2 (PDB#1L9V) monomer or on homology models of AU-1 NSP2 or CH4 NSP2 monomers, which were created using UCSF Chimera^48^. The PDB files of the modeled structures are available upon request. Prior to performing the simulations, the complete structures were explicitly solvated with a three-point water model (TIP3P) in rhombic dodecahedron water box (solute-box distance of 1.0 nm) under periodic boundary conditions, with charges neutralized by chloride ions. The AMBER99SB-ILDN force field was used for all simulations^49^. Starting structures were energy minimized until convergence at Fmax < 1000 kJ/mol/nm. A 100-picosecond position-restrained NVT equilibration simulation was run for water relaxation at 300 K using a modified Berendsen (velocity rescaling) thermostat, followed by a 100-picosecond NPT equilibration simulation using the Parrinello-Rahman barostat for pressure coupling. After equilibration, an unrestrained 20-nanosecond NPT molecular dynamics simulation was run at 300 K. Three trajectories initiated with different random seeds were run for each protein structure. The RMSF of α-carbons from each of the three trajectories was calculated using the gmx rmsf command in GROMACS. B-factors (i.e., Debye-Waller factors) for each residue were calculated from the RMSF values using an established equation [B-factor = (8Π^2^/3) × (RMSF)^2^]^50, 51^. One-way ANOVA analyses were performed using StatPlus, and *p* values < 0.05 were considered to be statistically significant.

### Data availability

The datasets generated during and/or analyzed during the current study are available from the corresponding author upon request.

## Electronic supplementary material

Fig. S1
