# Supplementary material for: Genetic determinants restricting the reassortment of heterologous NSP2 genes into the simian rotavirus SA11 genome
Source: Sci Rep. 2017 Aug 24;7:9301. doi: 10.1038/s41598-017-08068-w (PMC5571167; doi:10.1038/s41598-017-08068-w)
Supplement: Supplementary file 1 — Fig. S1 [file 41598_2017_8068_MOESM1_ESM.pdf]

Supplemental Information

for

Genetic determinants restricting the reassortment of heterologous NSP2 genes into the simian rotavirus SA11 genome

Rebecca Mingo[1§], Shu Zhang[1§], Courtney P. Long[1,2], Leslie E.W. LaConte[1], and Sarah M. McDonald[1,3]*

[1]Virginia Tech Carilion School of Medicine and Research Institute, Roanoke, VA

[2]Translational Biology, Medicine, and Health Graduate Program, Roanoke, VA

[3]Department of Biomedical Sciences and Pathobiology, Virginia-Maryland College of Veterinary Medicine, Blacksburg, VA

[§]Authors contributed equally

*Corresponding author current affiliation and address:

Sarah M. McDonald

Wake Forest University, Department of Biology

Wake Downtown, 455 Vine Street, Winston-Salem, NC 27101

Phone: (336)758-5000; Email: mcdonasm@wfu.edu

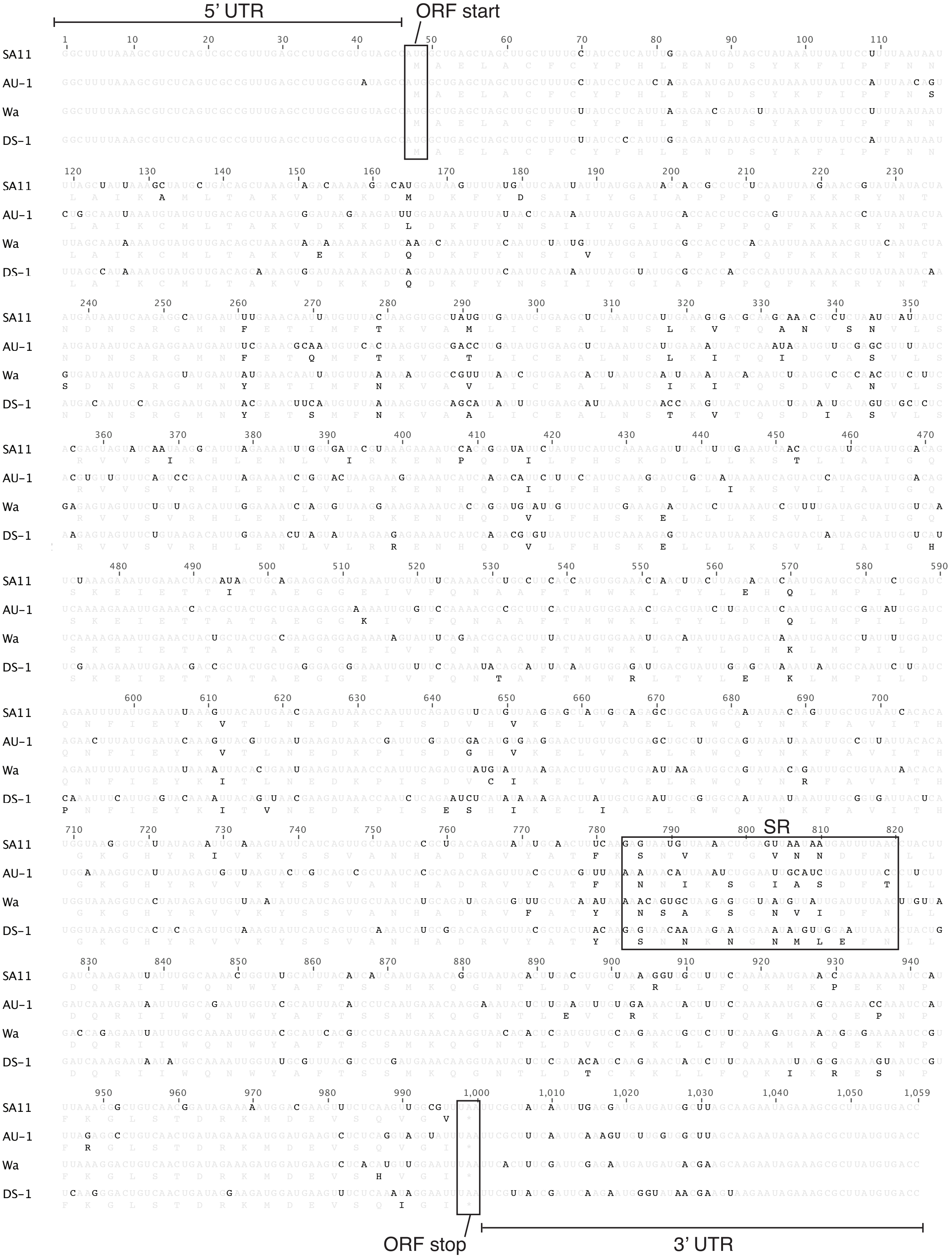

Fig. S1. Alignment of NSP2 genes/proteins for RV strains SA11, AU-1, Wa, and DS-1. Strain names are listed to the left of each nucleotide sequence and position numbers are listed on the top. Deduced amino acid sequences for NSP2 proteins are shown underneath the corresponding nucleotide sequence. Conserved residues are shown in grey and variable residues are shown in black. The locations of the UTRs are shown with brackets and labeled. The ORF start/stop codons and SR are outlined with black boxes and labeled.
